# Non-Coding RNAs to Aid in Neurological Prognosis after Cardiac Arrest

**DOI:** 10.3390/ncrna4040042

**Published:** 2018-12-18

**Authors:** Antonio Salgado-Somoza, Francesca Maria Stefanizzi, Pascal Stammet, David Erlinge, Hans Friberg, Niklas Nielsen, Yvan Devaux

**Affiliations:** 1 Cardiovascular Research Unit, Department of Population Health, Luxembourg Institute of Health, L-1445 Strassen, Luxembourg; antonio.salgadosomoza@lih.lu (A.S.-S.); francescamaria.stefanizzi@lih.lu (F.M.S.); 2 Medical and Health Department, Luxembourg Fire and Rescue Corps, L-2557 Luxembourg, Luxembourg; Pascal.Stammet@secours.etat.lu; 3 Department of Cardiology, Clinical Sciences, Lund University, 221 85 Lund, Sweden; david.erlinge@gmail.com; 4 Skane University Hospital, Lund University, 221 85 Malmo, Sweden; hans.a.friberg@gmail.com; 5 Helsingborg Hospital, Lund University, 25187 Helsingborg, Sweden; niklas.nielsen@telia.com

**Keywords:** cardiac arrest, biomarkers, non-coding RNAs

## Abstract

Cardiovascular disease in general, and sudden cardiac death in particular, have an enormous socio-economic burden worldwide. Despite significant efforts to improve cardiopulmonary resuscitation, survival rates remain low. Moreover, patients who survive to hospital discharge have a high risk of developing severe physical or neurological symptoms. Being able to predict outcomes after resuscitation from cardiac arrest would make it possible to tailor healthcare approaches, thereby maximising efforts for those who would mostly benefit from aggressive therapy. However, the identification of patients at risk of poor recovery after cardiac arrest is still a challenging task which could be facilitated by novel biomarkers. Recent investigations have recognised the potential of non-coding RNAs to aid in outcome prediction after cardiac arrest. In this review, we summarize recent discoveries and propose a handful of novel perspectives for the use of non-coding RNAs to predict outcome after cardiac arrest, discussing their use for precision medicine.

## 1. Background

Despite joint efforts from the clinical and research communities in the field, cardiovascular disease remains the leading cause of mortality in the modern world, accounting for 45% of all deaths in Europe [1]. A high percentage of these deaths is attributed to sudden death or cardiac arrest (CA). According to the Declaration of the European Parliament of 14 June 2012, some 400,000 people suffer a sudden out-of-hospital CA each year in Europe. Similar numbers can be found in the United States, where cardiac arrest affects more than 350,000 people per year [2].

## 2. Outcome after Cardiac Arrest

**Figure 1:**
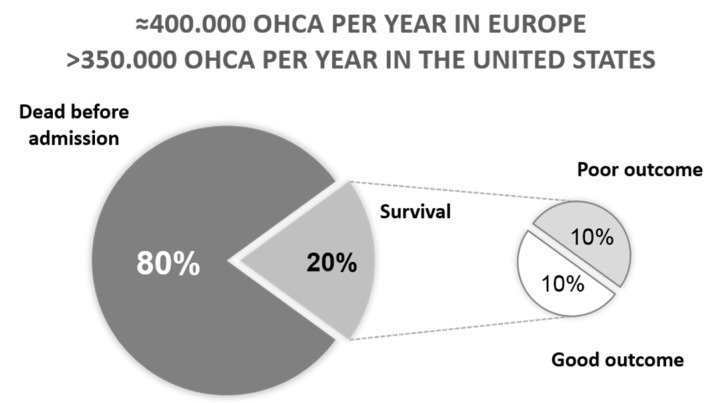
Graphic representation of the patient survival (showed as percentage) after Out-of-Hospital Cardiac Arrest (OHCA).

Outcome is extremely poor following CA, as more than half of the people who suffer an arrest will die before reaching the hospital. Overall, less than 10% of patients who receive cardiopulmonary resuscitation survive after out-of-hospital CA (Figure 1). The survival rate slightly ameliorates when CA occurs inside the hospital [3,4]. The expectancy of survival depends on both extrinsic (management shortly after the event or in the hospital) and intrinsic factors (gender, age…).

An important variable affecting the outcome after out-of-hospital CA (OHCA) is the application of proper and rapid cardiopulmonary resuscitation by bystanders [5,6]. The improvement of cardiopulmonary resuscitation techniques and the increased presence of automated external defibrillators in public areas are at least partly responsible for the tendency towards the better outcomes and survival rates observed in the past decades [4,7,8]. In addition, targeted temperature management at 33–36 °C has been implemented in clinical practice to protect the brain from ischemia-induced injury, but many questions about the ideal target temperature management remain unsolved [9]. As such, there is a need for new strategies to improve survival after OHCA.

Among patients who reach the hospital alive, approximately 50% survive in good condition (i.e., without major neurological sequelae [10]), whilst the other half suffer severe and irreversible neurological damage often leading to death [11], despite the use of modern, invasive, and intensive care treatment including targeted temperature management. In fact, in addition to hypothermia and early coronary reperfusion, patients in critical conditions need close monitoring of several parameters such as electrolytes and blood glucose. Mechanical ventilator supports are required to avoid hyperoxia and to ensure adequate ventilation. Pharmacological hemodynamic supports and devices are also needed in case of cardiogenic shock, mainly to improve arterial pressure and renal function. Lastly, a series of specific treatments are involved in case of other organ complications.

As mentioned above, several intrinsic factors affect the outcome after CA. For example, females are more prone to a worse outcome, which may be explained by differences in arrest circumstances, comorbidities, and in-hospital management [12]. In a large registry, men had a higher survival rate and a better neurological outcome at hospital discharge than women [13]. As for other cardiovascular diseases, survival expectancy after CA might be higher in obese patients and smokers (often called obesity and smoking paradoxes) [14,15,16], even though these are known risk factors triggering this cardiovascular complication. The delay between cardiac arrest and return of spontaneous circulation (ROSC), as well as the subsequent extent of brain damage, are both strongly associated with a poor outcome [17].

## 3. Prediction of Neurological Outcome after Cardiac Arrest

Being able to predict the outcome after CA would represent a breakthrough towards personalized/precision medicine. Extensive and costly treatments could be maximized to patients who are likely to survive without major neurological sequelae, and long futile care could be avoided in patients with severe and irreversible brain damage. The withdrawal of life-sustaining therapies could be adjudicated for patients with a certain poor prognosis. Importantly, patient relatives, who often experience a long and painful waiting period before being reliably informed of the prognosis, could be guided at an early stage [18]. As stated in the 2010 European Resuscitation Guidelines for Resuscitation, “a means of predicting neurological outcome that can be applied to individual patients immediately after ROSC is required” [19].

Current guidelines to predict outcome after CA recommend the combined use of neurophysiological tests, reflecting brain function, and brain-derived biomarkers released in the bloodstream after disruption of the blood-brain-barrier induced by cerebral ischemia, and hence, reflecting the extent of brain injury [20]. Clinical examination of the patient is of paramount importance for outcome prediction. The absence of normal ocular reflexes or response to pain and the presence of myoclonus during the first days after ROSC are indicators of poor prognosis. However, these markers can be attenuated or masked by sedation, making their interpretation difficult and unreliable. The absence of pupillary light and corneal reflexes on day 3 after CA has been reported to reliably predict poor outcome, with a false positive rate of 0% [21]. However, neurophysiological tests are limited by the need for specific expertise and training.

Box 1. **Main Protein biomarkers for CA prognosis: strengths and limitations.**

• **Neuron specific enolase (NSE):**

Strengths: Recognized as prognostic biomarker for CA in the guidelines of the American Heart Association [22].Blood levels are predictors of neurological outcome in a timeline going from 24 h to 72 h after ROSC [23,24,25,26].

Limitations: Increased levels are found in other disorders, leading to patient misclassification [27,28,29]. Haemolysis can increase serum levels of NSE. Outcome prediction earlier than 48 h shows lack of accuracy.

• **S100 calcium-binding protein B (S100B):**

Strengths: Predicts the ongoing of cerebral injury at a very early stage after CA [30,31].

Limitations: Lacks specificity and does not add value to the current prognostication models [26].

• **TAU protein:**

Strengths: High serum levels reveal axonal dysfunction. Increased circulating levels of the protein can be detected in patients after CA with a poor neurological outcome.

Limitations: Peaks of TAU are reached at 48 h and 96 h after ROSC [32,33]. Few data supporting its utility as marker of severity of acute brain injury after CA [32,33].

• **Plasma neurofilament light chain (NfL)**

Strengths: Sensitive marker of long-term outcome in patients after CA, its blood levels increase significantly and remain stable for 7 days after CA [34].

Limitations: Supporting evidence comes only from small single-centre patient cohorts.

• **Procalcitonin (PCT)**

Strengths: Its circulating levels increase significantly, within 2 h, during bacterial infections. Various small-cohort studies showed the PCT potential to predict poor outcome in CA patients with high accuracy [35,36,37].

Limitations: Confirmed biomarker for detection of acute infections and sepsis, which can act as confounding factors [38]. Larger studies needed to support its potential value as biomarker.

Neuron-specific enolase (NSE), the only biomarker so far recommended in the guidelines [20], needs serial assessment and high values in order to optimally predict poor outcomes [17,39]. In addition, there is no consensus on the threshold to be used as a definition of a high value, with suggestions ranging between 25–85 mg L^−1^ measured at different time points among studies [18,40]. Recently, Chung-Esaki and colleagues proposed the NSE ratio, instead of its absolute value, to track the protein changes over time after CA; however no threshold value has been identified, and future studies on larger cohorts need to be performed to validate the advantages of this method [41]. S100B is an acidic Ca^2+^-binding protein predominantly expressed in astroglial cells which is detected in the blood after CA and which has been reported as a potential biomarker of outcome [42,43]. Its use is limited by fluctuating levels due to peripheral injuries and to a short half-life of 25 min followed by renal clearance [44,45]. Inflammatory and cardiac markers such as interleukin-6 [46], procalcitonin [47], brain natriuretic peptides [48] and troponins [49] are also associated with outcome, but they are limited in accuracy (See Box 1). Lactate levels are increased in patients with poor outcome [50], and secretoneurin, a small peptide with a potential role in cardiomyocyte calcium homeostasis, improved the prediction of mortality and brain injury by NSE [51]. Yet, apart from NSE, only few of these markers are applied in clinical practice to help outcome prediction.

In summary, to date, no method securely predicts outcome after CA. To be clinically useful, a prognostication method requires a very low false positive rate (5% or lower), since it is undesirable to use a method which could predict a poor outcome in a patient who would eventually recover. Therefore, novel methods or biomarkers are needed to improve prediction of outcome after CA.

## 4. Non-Coding RNAs

Here, we focus on studies reporting potential benefits of non-coding RNA biomarkers on outcome prediction after CA (Table 1, Table 2 and Table 3). The explosion of RNA knowledge in recent years has provided the tools and opportunity to explore the potential of RNA molecules as biomarkers. Much attention has been brought to RNA molecules lacking protein-coding potential following the discovery that, although 80% of human genes are effectively transcribed into RNA molecules, only less than 2% of these molecules encode proteins [52]. These so-called non-coding RNAs are a subclass of the RNA family which contains multiple types of RNAs, classified according to their sub-cellular location, function, size and protein-coding potential.

### 4.1. MicroRNAs

MicroRNAs (miRNAs) are 20–22 nucleotides-long non-coding RNA molecules involved in gene regulation. Their role in heart development and disease as well as their biomarker potential to advance personalized medicine have been extensively studied [53]. However, the study of their role in CA is still in its infancy (Table 1). In a small proof-of-concept study including 28 patients after resuscitated CA, circulating levels of miR-122-5p and miR-21 were elevated in patients with poor outcome and were associated with neurological outcome and survival [54]. It is noteworthy that miR-21 was elevated in a murine model of traumatic brain injury [55], and plasma levels of miR-122-5p were increased in a porcine cardiogenic shock model and attenuated by hypothermia [56]. Those observations in animal models supported the results of the human small scale study. Lately, 23 microRNAs involved in neurologic, circulatory, metabolic, or vascular processes were studied in 45 patients and 15 controls from the Cardiac Arrest Blood Study. The vast majority of them were upregulated in sudden cardiac arrest when compared with age-, sex- and race-matched controls [57]. Among them, miR-122-5p presented higher expression values in successfully resuscitated patients compared to dead ones [57]. It has to be taken into account that miR-122-5p expression levels (as well as other miRNAs) could be influenced by other pathologies. Thus, a study involving 239 patients who suffered sudden cardiac death (SCD) showed lower hepatic expression of miR-122-5p and higher expression of miR-34a-5p in patients whose SCD was related to coronary artery disease (*n* = 68) compared with those with non coronary artery disease-related SCD (*n* = 27) [58]. When patients were stratified according to the level of liver damage, miR-122-5p presented the lowest expression values in patients with necroinflammatory statohepatitis, pointing at a deficit of this miRNA in the liver if this organ is subjected to an insult [58].

In another small-scale study, circulating levels of miR-124-3p, a brain-enriched miRNA, were elevated in patients with poor outcomes compared to patients with favourable outcomes [59]. Similarly to miR-21, miR-124-3p was previously described as a biomarker for ischemic brain injury, as demonstrated in an experimental model of cerebral stroke in rats [60]. It is known that circulating miRNAs can reflect brain damage, since dying neurons release microparticles carrying miRNAs in the cerebrospinal fluid [61]. As such, miR-124-3p arises as a perfect candidate to be measured in the bloodstream because its plasma levels are elevated in a rat model with a cerebral artery occlusion despite its neuron-specific origin [62]. Indeed, some miRNAs appear to be able to cross the blood brain barrier, which is disrupted following cerebral ischemia [63], and to accumulate in the blood.

Encouraging results from these small-scale studies motivated the testing of the association between circulating miRNAs and outcome after CA in larger cohorts. Consortium members of the Target Temperature Management (TTM)—trial (NCT01020916 [9]) investigated this association in plasma samples harvested 48h after CA from almost 600 CA patients. Using small RNA sequencing on 50 patients of the TTM cohort, miRNAs associated with neurological outcome, as assessed by the cerebral performance category score, were identified [64]. A follow-up validation study in the entire cohort revealed that among miRNA candidates, miR-124-3p was the most strongly associated with outcome. Together with demographic and clinical parameters, it was able to accurately predict neurological outcome (odds ratio of 1.62 with a 95% confidence interval of 1.13–2.32) and survival (hazard ratio of 1.63 with a 95% confidence interval of 1.37–1.93) [64]. Using the same TTM–trial cohort, miR-122-5p was able to provide an incremental predictive value to a model containing both clinical parameters and miR-124-3p, showing that combining multiple explanatory variables may increase the predictive value of miRNAs [65].

### 4.2. Long Non-Coding RNAs

Long non-coding RNAs (lncRNAs) are defined as RNA molecules longer than 200 nucleotides and lacking protein-coding potential [67,68]. The spreading of the use of deep RNA sequencing techniques has made possible the discovery and annotation of more than 146,000 lncRNAs in human (LNCipedia 4.1 [69]). Only a minority of these lncRNAs have known roles in pathophysiology, and even fewer have been shown to regulate cardiac development and disease [70,71,72,73]. This might be due in part to the relatively low conservation of lncRNAs between species which hinders mechanistic studies in animal models. While different mechanisms of action of lncRNAs have been proposed [70,72,73], much remains to be discovered regarding their regulation and function in cardiac disease. One interesting property of lncRNAs is their ability to bind and sequester miRNAs, acting as a sponge, and thereby inhibiting the action of miRNAs on gene expression.

There are some indications pointing at the potential of lncRNAs as biomarkers of cardiovascular disease [70,72,73]. Since lncRNA expression profiles in blood cells have been shown to be associated with outcome after acute myocardial infarction [74], and considering that the aetiology of CA is often a prior acute myocardial infarction, it can be expected that lncRNAs may also be associated with outcome after CA (Table 2).

LncRNAs are expressed in the brain and are involved in neurological disease development [75], thereby constituting candidate targets for future research. As an example, it has been shown that downregulation of the lncRNA Meg3 after 2 h of occlusion of the middle cerebral artery in rats reduced the lesion volume and improved neurological outcome through the stimulation of angiogenesis mediated by the Notch signalling pathway [76]. Silencing Meg3 in a human endothelial cell line induced an activation of pro-angiogenic properties of the cells, such as cell migration, sprouting and tube formation [76]. In hypertensive rats following 1h of occlusion, upregulation of FosDT modulates brain damage via an indirect modulation of downstream genes of repressor element-1 silencing transcription factor [77]. One of the downstream pathways of this transcription factor involves nuclear factor kappa B (NFκB) which is also associated with the lncRNAs C2dat1 and Gm4419. Indeed, C2dat1 regulates calmodulin kinase to promote neuronal survival after ischemia reperfusion in mice [78], while Gm4419 contributes to NFκB activation in rat microglial cells [79]. Furthermore, Gm4419 expression levels are upregulated in injured mouse astrocytes, and scavenge miR-466l-3p, which induces tumour necrosis factor alpha (TNFα) production and apoptosis [66]. The later study revealed an interaction of three different members of the RNA family (one lncRNA, one miRNA and one lncRNA), which shows the complex regulation networks taking place in the affected brain.

Maybe the best indication about the regulation of lncRNAs after CA arose from a rat model, where ventricular fibrillation was induced by alternating current. This study showed the upregulation of 37 lncRNAs and the downregulation of 21 lncRNAs in post-CA brain samples. [80]. Results from another study showed an upregulation of the lncRNA Neat1 after treatment with bexarotene (an agonist of the retinoid X receptor used in some types of cancer), thereby reducing inflammation and apoptosis [81]. This observation suggested that lncRNAs may be involved in a patient’s response to treatment. However, the study was carried out in different cellular and animal models whose relevance for humans needs further exploration. In addition, other attempts using animal models aimed to reflect how brain damage can alter the transcription of lncRNAs. In traumatic brain injury models in rodents, several lncRNAs, including the well-known Neat1 and Malat1, were regulated and considered as potential players in brain injury [82,83]. In a subarachnoid haemorrhage murine model, microarrays and deep RNA sequencing allowed researchers to identify more than 200 and 600 differentially-expressed lncRNAs, respectively [84,85]. Confirmation by PCR was achieved for several lncRNAs which were found to be either upregulated (MRuc008hvl and BC0092207) or downregulated after haemorrhage (XR_006756, MRAK017168, and MRAK038897) [84]. However, there is a lack of mechanistic studies following the discovery of the dysregulated RNAs by high throughput analysis. So, whether these lncRNAs actively contribute to brain injury or are mere bystanders remains to be determined.

In order to become useful biomarkers, lncRNAs would need to be measurable in the blood. As previously mentioned for miRNAs, the accumulation of lncRNAs in the blood will rely on their ability to cross the blood brain barrier. Whether lncRNAs have this capacity remains to be demonstrated.

### 4.3. Circular RNAs

A subclass of lncRNAs called circular RNAs (circRNAs), which are formed by a back-splicing event, showed a higher stability in body fluids compared to their linear counterparts [86,87]. Indeed, this circular form protects them from degradation by exoribonucleases, thus providing them with an interesting biomarker potential [88]. Although circRNAs have been proposed as candidate biomarkers of cardiovascular disease including heart failure [87], their presence in the blood has only been shown in blood cells [89], and whether they are able to cross the blood brain barrier remains to be determined. So far, there is no study directly linking the regulation of circRNAs with the prognosis after CA (Table 3).

Again, studies performed with animal models suggest a possible involvement of circRNAs in the underlying mechanisms of brain injury, be it ischemic or traumatic. Using a mouse model of stroke induced by occlusion of the middle cerebral artery, two microarray approaches using RNAse R treated RNA identified modified circRNAs patterns in the brain. Thus, after 45 min of ischemia and 48h of reperfusion, 1027 circRNAs were differentially expressed (914 upregulated and 113 downregulated) [90]. Among the differentially expressed RNAs, circRNA_40001, circRNA_013120, circRNA_25329 (upregulated), and circRNA_40806 (downregulated) were confirmed by PCR. When the mice were submitted to 90 min of ischemia followed by 6, 21 or 24 h of reperfusion, 283 circRNAs were altered in at least one time point, and 16 of them were common to all three time-points [91]. The top three upregulated (circ_008018, circ_015350, and circ_016128) and downregulated (circ_011137, circ_001729, and circ_006696) circRNAs were confirmed by PCR [91].

A microarray analysis revealed 98 upregulated and 94 downregulated circRNAs in a traumatic brain injury model [92], where four of them were confirmed by PCR (circ_006508 and circ_010705 were up-regulated whilst circ_001167 and circ_001168 were down-regulated). CircRNA dysregulation was studied in exosomes from brain explants of mouse with traumatic brain injury [93]. Next generation sequencing followed by a specific bioinformatics pipeline for the analysis of circRNAs identified 155 up- and 76 down-regulated circRNAs [93]. Therefore, the circular RNA reservoir might constitute another source of interesting biomarkers of outcome after CA.

## 5. Future Directions and Conclusions

The following points could be considered in future studies on the biomarker potential of non-coding RNAs after CA.

- Further deep RNA sequencing experiments are needed to identify the best candidates for prediction among the currently-identified 2500 human mature miRNAs, 146,000 human annotated lncRNAs and 32,000 predicted circRNAs.
- Independent validation of candidates in large patient cohorts will be the key to discovering robust and clinically-applicable biomarkers.
- Address gender specificities.
- Test the incremental predictive value of panels of non-coding RNAs using suitable correction strategies to avoid model overfitting.
- Assess the evolution of circulating levels of non-coding RNAs within the few hours/days after CA.
- Assess the influence of co-variates, such as age and target temperature, on circulating levels of non-coding RNAs.
- Define optimized protocols for blood sample collection, handling, storage and processing for RNA biomarkers assessment.
- Design molecular diagnostic assays for RNA assessment at the bedside that will allow clinically-applicable decision support systems combining biomarker assessment, neurophysiology, clinical examination, statistical analysis and risk stratification models.

Recent studies support the use of circulating miRNAs as predictors of outcome after CA. The potential of lncRNAs and circRNAs to aid in outcome prediction after CA needs to be further investigated. Using circulating, non-coding RNAs to risk stratify patients after CA would allow us to tailor healthcare to the individual patient, maximising efforts to those who would mostly benefit from aggressive therapy while avoiding therapeutic obstinacy in patients with poor prognoses. This would represent a major step forward towards precision medicine.

## Figures and Tables

**Table 1:** Current knowledge of microRNAs in cardiac arrest or related brain disease.

ID	Specie	Disease	Experimental Model	Observation	Ref.
miR-21	Human	OHCA	---	Elevated plasma levels in patients with poor neurological outcome	[54]
	Rat	TBI	Fluid percussion injury	Elevated serum levels in rats with poor outcome	[55]
miR-34a	Human	SCD	Coronary artery and non-alcoholic fatty liver disease	Higher hepatic levels in coronary artery disease-related SCD	[58]
miR-122	Human	OHCA	---	Elevated serum levels in patients with poor neurological outcome	[54,65]
		SCA	Ventricular tachycardia-derived cardiac arrest	Elevated in plasma from patients compared to controls. Elevated in successfully resuscitated or discharged alive only versus patients died in the field.	[57]
		SCD	Coronary artery and non-alcoholic fatty liver disease	Lower hepatic levels in coronary artery disease-related SCD	[58]
	Pig	Cardiogenic shock	LAD artery occlusion	Elevated plasma levels after injury. Attenuation by hypothermia.	[56]
miR-124	Human	OHCA	---	Elevated serum and plasma levels in patients with poor neurological outcome	[59,64]
	Rat	Ischemic brain damage	MCAO	Plasma biomarker of ischemic brain damage	[60,62]
miR-466l-3p	Mouse	Mechanical injury	Primary astrocytes	Inhibited *Tnfα* expression after stretch injury and interacted with lncRNA *Gm4419*	[66]
Other	Human	SCA	Ventricular tachycardia-derived cardiac arrest	Expression levels of plasmatic miRs were higher (n = 17) or lower (n = 3) in CA patients compared to controls. Mir-122 and miR-205 were elevated in patients successfully resuscitated versus death in the field. Lower levels of cardiac enriched microRNAs were observed in patients discharged alive versus the ones who died in the field.	[57]

CA: Cardiac Arrest; LAD: left anterior descending; MCAO: middle cerebral artery occlusion; OHCA: out-of-hospital cardiac arrest; SCA: Sudden Cardiac Arrest; SCD: Sudden Cardiac Death; TBI: traumatic brain injury.

**Table 2:** Current knowledge of lncRNAs in CA-related brain disease models.

ID	Specie	Disease	Experimental Model	Observation	Ref.
C2dat1	Mouse	Ischemic brain damage	MCAO	Upregulated after transient focal ischemia	[78]
FosDT	Rat	Ischemic brain damage	MCAO in spontaneous hypertensive rats	Increased after ischemic brain injury. Potentially regulated brain damage by association with key elements of the *Rest* complex, upstream of *NFκB*	[77]
Gm4419	Rat	Ischemia	OGD/R in primary microglial cells	Controled inflammatory response through *NFκB* signaling pathway	[79]
	Mouse	Mechanical injury	Primary astrocytes	Induced after stretch injury. Upregulates *Tnfα* expression by sponging miR-466l-3p	[66]
Meg3	Human	---	HMEC-1	Downregulation of MEG3 increased angiogenesis	[76]
	Rat	Ischemic brain damage	MCAO	Downregulated after ischemic stroke. Silencing of *Meg3* improved neurological outcome.	[76]
Neat1	Mouse	TBI	Controlled cortical impact	Upregulated after injury. Absence of Neat1 increases apoptosis around the impacted area.	[81,82]
	Mouse	Ischemia	OGD in primary new-born neurons, HT22, and BV2 lines	Upregulated under bexatorene treatment or OGD. Promoted axonal extension in primary neurons. Anti-inflammatory effect via *Pidd1*.	[81]
Other	Rat	CA-ROSC	Electrically-induced ventricular tachycardia followed by manual chest compression	Dysregulation of 58 lncRNAs and 258 mRNAs in brain cortex of rats.	[80]
		TBI	Fluid percussion injury	Upregulation of 271 lncRNAs in the hippocampus assessed by microarray, including 4 lncRNAs validated by PCR (*Zfas1*, *Bsr*, *Gas5*, and *Snhg6*)	[83]
		Stroke	Subarachnoid haemorrhage	Microarray analysis showed 64 upregulated and 144 downregulated lncRNAs between control and haemorrhagic animals.	[84]
	Mouse	TBI	Controlled cortical impact	Alteration of the expression levels of 823 lncRNAs assessed by RNA-Seq 24 h after injury.	[82]
		Stroke	Subarachnoid haemorrhage	RNA-Seq analysis identified 103 upregulated and 514 downregulated lncRNAs between injured and control mice.	[85]

BV2: microglial cell line. CA-ROSC: Cardiac Arrest-Return to Spontaneous Circulation; HMEC-1: human microvascular endothelial cells; HT22: hippocampal neuron line MCAO: middle cerebral artery occlusion; OGD/R: oxygen and glucose deprivation/reoxygenation; PCR: Polymerase Chain Reaction; TBI: traumatic brain injury.

**Table 3:** Current knowledge of circRNAs in brain disease animal models.

Specie	Disease	Model	Observation	Ref.
Mouse	Ischemic brain damage	MCAO	Microarray analysis after RNAse R treatment	[90]
			Microarray analysis after RNAse R treatment	[91]
	TBI	Fluid percussion injury	RNA sequencing analysis from brain exosomes.	[93]
Rat	TBI	Fluid percussion injury	Microarray analysis using RNA from ipsilateral hippocampus after RNAse R treatment	[92]

MCAO: middle cerebral artery occlusion; PCR: Polymerase Chain Reaction; TBI: traumatic brain injury.

## Abreviations

CA	Cardiac arrest
circRNAs	Circular RNAs
HUVEC	Human umbilical vein endothelial cells
LAD	Left anterior descending
lncRNA	Long non-coding RNA
MCAO	Middle cerebral artery occlusion
miRNA	MicroRNA
NFκB	Nuclear Factor kappa B
NSE	Neuron Specific Enolase
OGD/R	Oxygen and glucose deprivation/reoxygenation
OHCA	Out-of-hospital cardiac arrest
RNA	Ribonucleic acid
RNase	Ribonuclease
ROSC	Return Of Spontaneus Circulation
TBI	Traumatic brain injury
TNF	Tumor Necrosis Factor
TTM	Target Temperature Management
